# Supplementary material for: Molecular Mapping to Discover Reliable Salinity-Resilient QTLs from the Novel Landrace Akundi in Two Bi-Parental Populations Using SNP-Based Genome-Wide Analysis in Rice
Source: Int J Mol Sci. 2023 Jul 6;24(13):11141. doi: 10.3390/ijms241311141 (PMC10342095; doi:10.3390/ijms241311141)
Supplement: Supplementary file 1 [file ijms-24-11141-s001.zip › ijms-2445956-supplementary/ijms-2445956-revised-supplementary/Supplementary Table S1.pdf]

Supplementary Table S1: List of SNP markers (SNP ID) used in the QTL analysis

SNP Code	SNP Name	SNP Code	SNP Name	SNP Code	SNP Name
M1	chr01_194844	M24	chr01_9164508	M47	chr01_16596849
M2	chr01_375814	M25	chr01_10100605	M48	chr01_17008280
M3	chr01_717702	M26	chr01_10202227	M49	chr01_17219764
M4	chr01_932866	M27	chr01_10645259	M50	chr01_17426109
M5	chr01_1044946	M28	chr01_10846090	M51	chr01_18102213
M6	chr01_1175585	M29	chr01_11250486	M52	chr01_18332445
M7	chr01_1532281	M30	SALTOL-AUS	M53	chr01_18501890
M8	chr01_2100471	M31	SALTOL-ARO	M54	chr01_19008526
M9	chr01_2277961	M32	chr01_12216652	M55	chr01_19308987
M10	chr01_2532252	M33	chr01_12335190	M56	chr01_20262103
M11	chr01_2726468	M34	chr01_12809255	M57	chr01_20418175
M12	chr01_2865232	M35	chr01_12941687	M58	chr01_20562598
M13	chr01_3236648	M36	chr01_13442034	M59	chr01_21011077
M14	chr01_3529691	M37	chr01_13777831	M60	chr01_22554065
M15	chr01_4167976	M38	chr01_13902629	M61	IRGSP1_C01_22891448
M16	chr01_4307004	M39	chr01_14375630	M62	chr01_23091103
M17	chr01_5064126	M40	chr01_14508449	M63	chr01_23240729
M18	chr01_5510350	M41	chr01_14937939	M64	chr01_23656773
M19	chr01_6130240	M42	chr01_15092392	M65	IRGSP1_C01_23735018
M20	chr01_6543217	M43	chr01_15393352	M66	chr01_24050118
M21	chr01_7068516	M44	chr01_15883941	M67	chr01_24722142
M22	chr01_7563923	M45	chr01_16113201	M68	chr01_26187514

M23	chr01_8760562	M46	chr01_16284866	M69	chr01_27046296
**SNP Code**	**SNP Name**	**SNP Code**	**SNP Name**	**SNP Code**	**SNP Name**
M70	chr01_27330724	M93	chr01_37680628	M116	chr02_890901
M71	chr01_28142020	M94	chr01_37920337	M117	DTY2-2_1
M72	chr01_28478660	M95	DTY1-1_1	M118	MS-IR36
M73	chr01_28826738	M96	DTY1-1_2	M119	chr02_1082416
M74	chr01_30569798	M97	chr01_38632196	M120	chr02_1380527
M75	chr01_31396842	M98	DTY1-1_3	M121	chr02_1487009
M76	chr01_32041358	M99	SCT1_1	M122	chr02_1660713
M77	chr01_32359998	M100	SCT1_2	M123	chr02_2008370
M78	chr01_32563460	M101	QSES1-2_1	M124	chr02_2334960
M79	chr01_32766139	M102	chr01_39548506	M125	DTY2-2_2
M80	chr01_33164035	M103	QSES1-2_2	M126	chr02_2819095
M81	chr01_33472174	M104	chr01_39955529	M127	chr02_2983451
M82	chr01_33695501	M105	QSES1-2_4	M128	chr02_3136771
M83	chr01_33901317	M106	chr01_40699488	M129	chr02_3477202
M84	chr01_34201543	M107	chr01_41020155	M130	chr02_3830712
M85	chr01_34363755	M108	chr01_41911439	M131	chr02_4047646
M86	chr01_34508997	M109	chr01_42425574	M132	chr02_4342883
M87	chr01_35259107	M110	chr01_42877440	M133	IRGSP1_C02_4549475
M88	chr01_35589637	M111	chr01_43074709	M134	chr02_4823175
M89	chr01_35794891	M112	chr02_103273	M135	DTY2-2_3
M90	chr01_36150523	M113	chr02_316859	M136	chr02_4930742
M91	chr01_36419842	M114	chr02_494373	M137	chr02_5836334

M92	chr01_37274755	M115	chr02_629512	M138	chr02_6373446
**SNP Code**	**SNP Name**	**SNP Code**	**SNP Name**	**SNP Code**	**SNP Name**
M139	chr02_6805305	M162	chr02_20824958	M185	chr02_29568762
M140	chr02_7238793	M163	chr02_21060669	M186	chr02_29718772
M141	chr02_7342223	M164	chr02_21832777	M187	chr02_30345735
M142	chr02_8019748	M165	chr02_22340836	M188	chr02_30530432
M143	chr02_8814718	M166	chr02_23073414	M189	chr02_31135814
M144	chr02_9570228	M167	IRGSP1_C02_23246863	M190	chr02_31403314
M145	chr02_9801099	M168	chr02_23396915	M191	chr02_31678155
M146	chr02_9975660	M169	chr02_23538743	M192	chr02_31914486
M147	HIS1	M170	chr02_23852677	M193	chr02_32101050
M148	chr02_10555263	M171	chr02_24168273	M194	chr02_32303539
M149	chr02_10874369	M172	chr02_24264653	M195	chr02_33035683
M150	chr02_11701123	M173	chr02_24772301	M196	chr02_34072964
M151	chr02_13962476	M174	chr02_24887320	M197	chr02_34425408
M152	chr02_16464872	M175	chr02_25193353	M198	chr02_34707946
M153	chr02_16919033	M176	chr02_25415397	M199	IRGSP1_C02_34852782
M154	chr02_17160895	M177	chr02_25949636	M200	chr02_34868096
M155	chr02_17497082	M178	chr02_26406867	M201	chr02_35216781
M156	chr02_17789129	M179	chr02_27548893	M202	chr02_35401891
M157	chr02_18370293	M180	chr02_28049479	M203	chr02_35568365
M158	chr02_18811812	M181	chr02_28211032	M204	chr02_35818319
M159	chr02_19642635	M182	chr02_28778297	M205	chr03_484812
M160	chr02_19981768	M183	chr02_29011747	M206	chr03_851785

M161	chr02_20145859	M184	chr02_29415200	M207	DTY3-2-SWARNA_1
**SNP Code**	**SNP Name**	**SNP Code**	**SNP Name**	**SNP Code**	**SNP Name**
M208	DTY3-2-IR64_1	M231	chr03_9636224	M254	chr03_19929911
M209	DTY3-2-N22_1	M232	chr03_9751213	M255	chr03_20664396
M210	chr03_1270943	M233	chr03_9878813	M256	chr03_21003404
M211	chr03_1481412	M234	chr03_10184356	M257	chr03_22050510
M212	chr03_1761412	M235	chr03_10304250	M258	chr03_22633713
M213	chr03_2151496	M236	chr03_10544320	M259	chr03_23037368
M214	chr03_2885423	M237	chr03_10798982	M260	chr03_23354868
M215	chr03_3382980	M238	chr03_11644944	M261	chr03_23851835
M216	chr03_3657643	M239	chr03_11851886	M262	chr03_24413888
M217	chr03_4047654	M240	chr03_12564300	M263	chr03_24682521
M218	chr03_4321231	M241	chr03_13224089	M264	chr03_25157655
M219	chr03_5109298	M242	chr03_13932387	M265	chr03_25259336
M220	chr03_5880211	M243	chr03_14169727	M266	chr03_25530777
M221	chr03_6283461	M244	chr03_14554651	M267	chr03_25833182
M222	chr03_6902921	M245	IRGSP1_C03_14933451	M268	chr03_26334305
M223	chr03_7200676	M246	chr03_15072791	M269	chr03_26456796
M224	chr03_7605347	M247	chr03_15365726	M270	chr03_26982172
M225	IRGSP1_C03_7752789	M248	GS3	M271	chr03_27275567
M226	chr03_7798537	M249	chr03_17307455	M272	chr03_27494934
M227	chr03_8532555	M250	chr03_17827706	M273	chr03_27813704
M228	chr03_8711509	M251	chr03_18273682	M274	chr03_28851199
M229	chr03_9026803	M252	chr03_18629240	M275	chr03_29152266

M230	chr03_9375374	M253	chr03_19667207	M276	chr03_29430694
**SNP Code**	**SNP Name**	**SNP Code**	**SNP Name**	**SNP Code**	**SNP Name**
M277	chr03_30247826	M300	IRGSP1_C04_602842	M323	chr04_10991011
M278	chr03_30604896	M301	DTY4-1_2	M324	chr04_11645506
M279	DTY3-1_2	M302	chr04_1022225	M325	chr04_11879947
M280	chr03_31025197	M303	chr04_1136986	M326	chr04_12594753
M281	chr03_31190989	M304	chr04_1719865	M327	chr04_12665796
M282	DTY3-1_4	M305	DTY4-1_3	M328	chr04_12684621
M283	chr03_31427789	M306	chr04_3411505	M329	chr04_12711415
M284	chr03_31614921	M307	IRGSP1_C04_4319691	M330	chr04_12955347
M285	chr03_32063693	M308	chr04_4456304	M331	chr04_13551673
M286	chr03_32430702	M309	chr04_5111222	M332	chr04_13665093
M287	chr03_32577650	M310	chr04_5526487	M333	chr04_14229856
M288	chr03_32832500	M311	chr04_6027557	M334	chr04_14814591
M289	chr03_33080942	M312	chr04_6596219	M335	chr04_15389648
M290	chr03_33713486	M313	chr04_6913215	M336	chr04_15944977
M291	chr03_34275405	M314	BPH17_1	M337	chr04_16289829
M292	chr03_34476308	M315	BPH17_3	M338	chr04_16715200
M293	chr03_34678981	M316	BPH17_2	M339	chr04_17465113
M294	chr03_35093761	M317	chr04_7170237	M340	chr04_17666183
M295	chr03_35636092	M318	chr04_7304413	M341	chr04_18136052
M296	chr03_35824355	M319	chr04_7871436	M342	chr04_18706317
M297	chr03_36206765	M320	chr04_8217877	M343	chr04_19123643
M298	DTY4-1_1	M321	chr04_10077188	M344	chr04_19353709

M299	chr04_193676	M322	chr04_10468507	M345	chr04_20507218
**SNP Code**	**SNP Name**	**SNP Code**	**SNP Name**	**SNP Code**	**SNP Name**
M346	chr04_21237749	M369	COLD1-JAP	M392	chr05_3092113
M347	chr04_21517348	M370	chr04_30438901	M393	chr05_3470087
M348	chr04_21864875	M371	chr04_30879875	M394	chr05_3817643
M349	chr04_22435296	M372	NAL1	M395	chr05_4205413
M350	chr04_22808095	M373	TBV1_2	M396	chr05_4589783
M351	chr04_23046635	M374	chr04_31509863	M397	chr05_4819475
M352	chr04_23248365	M375	chr04_32038496	M398	chr05_4953754
M353	chr04_23846299	M376	chr04_32189893	M399	GW5_1
M354	chr04_24061518	M377	chr04_33073892	M400	GW5_2
M355	RYMV1-5	M378	chr04_33448911	M401	chr05_6129755
M356	RYMV1-2	M379	chr04_34643455	M402	chr05_6544781
M357	chr04_25245272	M380	chr04_34925111	M403	chr05_6607675
M358	chr04_25437295	M381	chr04_35395197	M404	chr05_6833061
M359	chr04_26309089	M382	chr05_44806	M405	chr05_7195992
M360	chr04_26545849	M383	chr05_201193	M406	chr05_7328783
M361	chr04_26832471	M384	chr05_437499	M407	chr05_7706179
M362	chr04_27540988	M385	chr05_440644	M408	chr05_8080666
M363	chr04_28004788	M386	chr05_661832	M409	chr05_8384781
M364	chr04_28901886	M387	chr05_922530	M410	chr05_8585466
M365	chr04_29533215	M388	chr05_1487229	M411	chr05_9712988
M366	chr04_29715617	M389	chr05_1832604	M412	chr05_10539124
M367	chr04_29881066	M390	chr05_2045112	M413	chr05_10886331

M368	chr04_30174083	M391	chr05_2291156	M414	chr05_11679660
**SNP Code**	**SNP Name**	**SNP Code**	**SNP Name**	**SNP Code**	**SNP Name**
M415	chr05_13325546	M438	chr05_23143060	M461	WX-A_GROUP
M416	chr05_13768744	M439	chr05_23661597	M462	WX-A-RC222
M417	IRGSP1_C05_13854594	M440	chr05_24090514	M463	chr06_1768998
M418	chr05_14412355	M441	chr05_25234430	M464	chr06_2430760
M419	chr05_14928105	M442	chr05_27086886	M465	chr06_2535102
M420	chr05_16262586	M443	chr05_27531788	M466	chr06_2783620
M421	chr05_16500527	M444	chr05_27627633	M467	chr06_2808467
M422	chr05_16752221	M445	chr05_27899049	M468	chr06_3140863
M423	chr05_16955234	M446	chr05_28101376	M469	chr06_3244503
M424	chr05_17012134	M447	chr05_28344771	M470	chr06_3431721
M425	chr05_17017106	M448	chr05_28772543	M471	chr06_3598843
M426	chr05_18405433	M449	chr05_29100308	M472	chr06_3632725
M427	chr05_18816269	M450	chr06_244274	M473	chr06_3721346
M428	chr05_19098650	M451	chr06_400753	M474	chr06_3951547
M429	chr05_19155333	M452	chr06_646915	M475	chr06_4076903
M430	chr05_19516545	M453	chr06_677288	M476	chr06_4641044
M431	chr05_19723342	M454	BPH32	M477	chr06_4757948
M432	chr05_19974373	M455	chr06_1272777	M478	chr06_5195334
M433	chr05_20331215	M456	chr06_1378891	M479	chr06_5707236
M434	chr05_20551103	M457	chr06_1521855	M480	chr06_5894169
M435	chr05_21494622	M458	chr06_1768006	M481	chr06_6093491
M436	chr05_22295097	M459	WX-OP	M482	chr06_6508525

M437	chr05_22711811	M460	WX-INT	M483	SSIIA-3B
SNP Code	SNP Name	SNP Code	SNP Name	SNP Code	SNP Name
M484	chr06_7629011	M507	chr06_20462203	M530	chr06_29208264
M485	chr06_8153078	M508	chr06_21246829	M531	chr06_29416997
M486	chr06_8405025	M509	chr06_21491695	M532	chr06_30809492
M487	chr06_9222038	M510	chr06_21818860	M533	chr07_514293
M488	chr06_9836381	M511	chr06_22371041	M534	chr07_770090
M489	chr06_10006906	M512	chr06_24003856	M535	chr07_940832
M490	PI9_1	M513	TGW6	M536	chr07_1058718
M491	chr06_10418594	M514	chr06_25277863	M537	chr07_1195861
M492	chr06_10966744	M515	chr06_26139965	M538	chr07_1317761
M493	chr06_11131403	M516	chr06_26513610	M539	chr07_1432567
M494	chr06_11692013	M517	chr06_26938344	M540	chr07_1558687
M495	chr06_12989252	M518	chr06_27275515	M541	chr07_1865306
M496	chr06_13702743	M519	chr06_27364485	M542	chr07_2128881
M497	chr06_14006726	M520	chr06_27627615	M543	chr07_2331583
M498	chr06_14504992	M521	chr06_27761109	M544	chr07_4481788
M499	chr06_15358704	M522	XA7_1	M545	chr07_5442983
M500	chr06_15538142	M523	XA7_2	M546	chr07_5729056
M501	chr06_16304717	M524	chr06_28100960	M547	chr07_6029723
M502	chr06_16614854	M525	chr06_28107682	M548	chr07_7317122
M503	chr06_16806254	M526	XA7_3	M549	chr07_8140854
M504	chr06_17076390	M527	chr06_28201282	M550	chr07_8320872
M505	chr06_17698448	M528	chr06_28723997	M551	chr07_8487862

M506	chr06_19989362	M529	chr06_29056693	M552	GHD7-4
SNP Code	SNP Name	SNP Code	SNP Name	SNP Code	SNP Name
M553	chr07_11290725	M576	AG3_2	M599	chr08_3866523
M554	chr07_13639513	M577	chr07_24194267	M600	chr08_4190544
M555	chr07_14729119	M578	chr07_24313817	M601	DTH8-IR24
M556	chr07_15340454	M579	chr07_24507157	M602	chr08_4511882
M557	IRGSP1_C07_16037152	M580	chr07_25339609	M603	chr08_4853081
M558	chr07_16313631	M581	chr07_25935801	M604	GM4_1
M559	IRGSP1_C07_19408599	M582	chr07_27021585	M605	chr08_5394572
M560	chr07_17654305	M583	chr07_27883987	M606	GM4_3
M561	chr07_17877865	M584	chr07_28187994	M607	GM4_4
M562	chr07_18208798	M585	chr07_28409912	M608	chr08_5634800
M563	chr07_18503646	M586	chr07_29062014	M609	PI33_1
M564	chr07_18689329	M587	chr08_213580	M610	chr08_6269190
M565	chr07_18895399	M588	chr08_658032	M611	PI33_2
M566	chr07_20514892	M589	chr08_811201	M612	PI33_3
M567	chr07_21135004	M590	chr08_1088007	M613	chr08_7854002
M568	chr07_21539524	M591	chr08_1198055	M614	chr08_7950638
M569	TSV1_UM	M592	chr08_1836529	M615	chr08_8386031
M570	chr07_22277144	M593	chr08_2208785	M616	chr08_8580913
M571	chr07_22593213	M594	chr08_2673447	M617	chr08_8703041
M572	chr07_23087994	M595	chr08_2878924	M618	chr08_8897653
M573	AG3_1	M596	chr08_3212821	M619	chr08_9289473
M574	chr07_23535052	M597	chr08_3365127	M620	chr08_9769758

M575	chr07_23785728	M598	chr08_3682006	M621	chr08_10105233
SNP Code	SNP Name	SNP Code	SNP Name	SNP Code	SNP Name
M622	chr08_10188041	M645	chr08_17618902	M668	chr08_26709228
M623	chr08_10358484	M646	chr08_18108496	M669	XA13_2
M624	IRGSP1_C08_10441781	M647	chr08_18221884	M670	chr08_26755059
M625	chr08_11248949	M648	chr08_18552226	M671	chr08_26898822
M626	chr08_11370680	M649	chr08_18719247	M672	chr08_27039158
M627	chr08_11565636	M650	chr08_19351882	M673	IRGSP1_C08_27133000
M628	chr08_11727432	M651	chr08_19833397	M674	chr08_27282645
M629	chr08_11888511	M652	chr08_20053642	M675	chr08_27563586
M630	chr08_12145970	M653	chr08_21011651	M676	chr08_28125942
M631	chr08_12421145	M654	chr08_21253397	M677	chr09_148594
M632	chr08_12723619	M655	chr08_21667240	M678	chr09_496013
M633	chr08_13017690	M656	chr08_22252502	M679	chr09_591085
M634	chr08_13354771	M657	chr08_22776414	M680	chr09_1098249
M635	chr08_13741571	M658	chr08_23713744	M681	chr09_1467951
M636	chr08_13950899	M659	chr08_24125399	M682	chr09_2096551
M637	chr08_14184612	M660	chr08_24205833	M683	chr09_2298381
M638	chr08_14676962	M661	chr08_24753844	M684	chr09_2380203
M639	chr08_14799421	M662	IRGSP1_C08_24853648	M685	chr09_2930321
M640	chr08_14986111	M663	WFP1	M686	chr09_3357568
M641	chr08_15439243	M664	chr08_25467581	M687	chr09_3603443
M642	chr08_16618829	M665	chr08_25757847	M688	chr09_3934768
M643	chr08_16799413	M666	PGWC8-2	M689	chr09_4136552

M644	chr08_17319167	M667	chr08_26448560	M690	chr09_4347203
SNP Code	SNP Name	SNP Code	SNP Name	SNP Code	SNP Name
M691	chr09_5008706	M714	AG1_1	M737	chr10_790044
M692	chr09_5305969	M715	chr09_12326525	M738	chr10_1562931
M693	chr09_5812738	M716	chr09_14920092	M739	chr10_2129424
M694	chr09_5922125	M717	chr09_15322775	M740	chr10_2258418
M695	chr09_6093984	M718	chr09_15429734	M741	chr10_3358419
M696	IRGSP1_C09_6205891	M719	DEP1-1	M742	chr10_3400212
M697	chr09_6252407	M720	DEP1-2	M743	chr10_4019997
M698	chr09_6796269	M721	chr09_16616509	M744	chr10_4281052
M699	chr09_6913547	M722	chr09_17213519	M745	chr10_5257224
M700	chr09_7116276	M723	chr09_17447522	M746	chr10_5488256
M701	chr09_7280339	M724	chr09_17707080	M747	chr10_5826161
M702	chr09_8511436	M725	chr09_19322095	M748	chr10_6648158
M703	chr09_8851945	M726	chr09_19683788	M749	chr10_6798684
M704	chr09_9252104	M727	chr09_20138174	M750	chr10_7120214
M705	chr09_9626056	M728	chr09_20682114	M751	chr10_7397104
M706	chr09_9945591	M729	chr09_21148430	M752	chr10_7530092
M707	chr09_10119114	M730	chr09_21348882	M753	chr10_8404652
M708	chr09_10324014	M731	chr09_21612758	M754	chr10_8795028
M709	chr09_10757351	M732	chr09_21667022	M755	chr10_9095431
M710	chr09_11237877	M733	chr09_21885499	M756	chr10_10476379
M711	chr09_11746684	M734	chr09_21896910	M757	chr10_10755400
M712	chr09_11848827	M735	chr09_22076185	M758	chr10_11195773

M713	chr09_12029697	M736	chr10_146531	M759	chr10_11664750
SNP Code	SNP Name	SNP Code	SNP Name	SNP Code	SNP Name
M760	chr10_11918679	M783	chr10_19678268	M806	chr11_9933184
M761	IRGSP1_C10_12035291	M784	chr10_19899160	M807	chr11_10741559
M762	chr10_12145295	M785	chr10_21338558	M808	chr11_11715177
M763	chr10_12658334	M786	chr10_21596449	M809	chr11_12142966
M764	chr10_12780901	M787	chr11_256688	M810	chr11_12583306
M765	chr10_13551338	M788	chr11_650148	M811	chr11_13020856
M766	chr10_13778768	M789	chr11_2393690	M812	chr11_13926471
M767	chr10_14086902	M790	chr11_2875870	M813	chr11_14737362
M768	chr10_14640458	M791	chr11_3516253	M814	chr11_15447862
M769	chr10_14777004	M792	chr11_3802930	M815	chr11_15638505
M770	chr10_15824876	M793	chr11_4783262	M816	chr11_15850522
M771	chr10_16229530	M794	chr11_5615885	M817	chr11_16284824
M772	chr10_16742656	M795	chr11_5852961	M818	chr11_17047762
M773	chr10_17397576	M796	chr11_6088667	M819	chr11_17292835
M774	chr10_18098744	M797	chr11_6472086	M820	chr11_17489208
M775	chr10_18302926	M798	chr11_7542820	M821	chr11_17697337
M776	chr10_18583084	M799	chr11_8083891	M822	chr11_17908099
M777	RF4	M800	chr11_8247118	M823	STV11
M778	chr10_18852690	M801	chr11_8601502	M824	SWEET14_PRO1
M779	chr10_19025553	M802	chr11_8786491	M825	SWEET14_2
M780	chr10_19217152	M803	chr11_9183139	M826	chr11_18290104
M781	chr10_19378143	M804	chr11_9413614	M827	chr11_18936070

M782	chr10_19421322	M805	chr11_9561302	M828	chr11_18993024
SNP Code	SNP Name	SNP Code	SNP Name	SNP Code	SNP Name
M829	chr11_19648542	M852	chr11_24086107	M875	chr12_2324105
M830	chr11_19898835	M853	chr11_24100905	M876	chr12_2651274
M831	chr11_20074439	M854	chr11_24585862	M877	chr12_3086293
M832	chr11_20239426	M855	chr11_25135188	M878	chr12_3217524
M833	chr11_20647651	M856	PI54_5	M879	chr12_3285571
M834	chr11_20837771	M857	chr11_25279481	M880	chr12_3544726
M835	chr11_21190115	M858	chr11_25432067	M881	chr12_4141420
M836	chr11_21446099	M859	chr11_25811598	M882	chr12_4433511
M837	chr11_21689303	M860	chr11_26300403	M883	chr12_4554103
M838	chr11_21691103	M861	IRGSP1_C11_27391141	M884	chr12_5041059
M839	chr11_22044151	M862	IRGSP1_C11_27571141	M885	chr12_7114076
M840	XA23_2	M863	chr11_27603799	M886	chr12_7445812
M841	IRGSP1_C11_22303236	M864	chr11_27817978	M887	chr12_7917509
M842	chr11_22440795	M865	chr11_27994133	M888	chr12_8070782
M843	chr11_22453819	M866	PIK_2	M889	chr12_8240608
M844	chr11_22562351	M867	PIK_1	M890	chr12_8794364
M845	PB1_1	M868	PIKH_2	M891	chr12_8913217
M846	chr11_22912310	M869	PIKH_1	M892	chr12_9120135
M847	chr11_23023708	M870	XA26_1	M893	chr12_9540170
M848	chr11_23080452	M871	chr11_28625316	M894	chr12_10051752
M849	chr11_23083051	M872	XA4_2	M895	chr12_10469146
M850	chr11_23231455	M873	chr11_28966693	M896	chr12_10509878

M851	IRGSP1_C11_23731426	M874	chr12_350921	M897	chr12_11257069
SNP Code	SNP Name	SNP Code	SNP Name	SNP Code	SNP Name
M898	chr12_11720114	M914	chr12_16591134	M930	chr12_23190179
M899	chr12_12075622	M915	chr12_16990670	M931	chr12_23387696
M900	chr12_12509917	M916	DTY12-1_2	M932	chr12_23828922
M901	chr12_12904225	M917	chr12_17443323	M933	chr12_24088209
M902	chr12_13248798	M918	chr12_17571574	M934	chr12_24145303
M903	chr12_13764151	M919	chr12_18003414	M935	chr12_24317071
M904	chr12_14060565	M920	chr12_18881059	M936	chr12_24417433
M905	chr12_14489210	M921	chr12_19161731	M937	chr12_24705450
M906	chr12_14599449	M922	chr12_19522102	M938	chr12_24913573
M907	chr12_14835375	M923	chr12_19578931	M939	chr12_25490919
M908	chr12_14936674	M924	chr12_19829588	M940	chr12_25756224
M909	chr12_15140411	M925	chr12_21446549	M941	chr12_26259494
M910	chr12_15342492	M926	chr12_21731719	M942	chr12_26463351
M911	IRGSP1_C12_15966147	M927	chr12_22600624	M943	chr12_26665728
M912	chr12_15960393	M928	chr12_23066809	M944	chr12_27023424
M913	chr12_16287347	M929	chr12_23108164	M945	chr12_27304351
