# Supplementary material for: Molecular Mapping to Discover Reliable Salinity-Resilient QTLs from the Novel Landrace Akundi in Two Bi-Parental Populations Using SNP-Based Genome-Wide Analysis in Rice
Source: Int J Mol Sci. 2023 Jul 6;24(13):11141. doi: 10.3390/ijms241311141 (PMC10342095; doi:10.3390/ijms241311141)
Supplement: Supplementary file 1 [file ijms-24-11141-s001.zip › ijms-2445956-supplementary/ijms-2445956-revised-supplementary/Supplementary Table S2.pdf]

Supplementary Table S2. Putative candidate genes in the QTL regions showing probable functions and references

Characters	QTL name	Chr.	Position (bp)	Number of locus	Candidate gene	Possible function	Reference
SES	*qSES1*	1	38723347-38724165	16	*LOC_Os01g66670*	expressed protein (drought-induced proteins, anther and pollen wall remodelling/metabolism proteins contribute to the tolerance of rice to salt stress)	Ke *et al.*(2009) Sarhadi *et al.*(2012)
Survival rate (%)	*qSUR11*	11	5357710-5359379	15	*LOC_Os11g09990*	mTERF family protein, expressed (coordinate mitochondrial transcription, growth, development and stress response)	Roberti *et. al.* (2010) Zhao *et al.* (2014)
Shoot length	*qSL1*	1	39794226-39799341	18	*LOC_Os01g68490*	tetratricopeptide-like helical, putative, expressed (abscisic acid responses and osmotic stress tolerance, enable plants to cope up with adverse environmental conditions)	Schapire *et al.*(2008) Sharma *et al.*(2016)
	qSL8	8	5613578-5616235	20	*LOC_Os08g09715*	F-box domain containing protein, expressed (F-box protein-encoding genes during floral transition as well as panicle and seed development, play a variety of roles in developmental processes including plant hormonal signal transduction, floral development, secondary metabolism, senescence, circadian rhythms, and responses to both biotic and abiotic stresses)	Jain *et al.* (2007) Zhang *et al.*(2019)

Characters	QTL name	Chr.	Position (bp)	Number of locus	Candidate gene	Possible function	Reference
Shoot dry weight	*qSDW1*	1	31473897-31477599	17	*LOC_Os01g54700*	retrotransposon protein, putative, Ty1-copia subclass, expressed (tuning gene expression during plant development salinity, played a major role in shaping genome structure).	Wang *et al.* (1999)
	qSDW10	10	17499639-17503204	12	*LOC_Os10g33310*	cyclin-dependent kinase inhibitor, putative, expressed (control cell growth, controlling cell cycle progression)	De *et al.*(2017) Vieira *et al.* (2017) Prabhu *et al.* (1997)
Root length	*qRL1*	1	10713139-10714271	16	*LOC_Os01g18950*	peroxidase precursor, putative, expressed (increases protection against oxidative stress, highly tolerant to different stresses allowing survival when water supply is a limiting factor).	Wang *et al.* (2011) Martínez *et al.*(2021)
SPAD value	*qSPAD7*	7	23314482-23319154	20	*LOC_Os07g38860*	OsGH3.10 - Probable indole-3-acetic acid-amidosynthetase, expressed	Ding *et al.*(2008)
Na^+ conc.	*qNa2*	2	34274401-34276956	17	*LOC_Os02g56010*	anthocyanidin 3-O-glucosyltransferase, putative, expressed	Fukuchi-Mizutani *et al.* (2003)
	qNa10	10	17547961-17548260	11	*LOC_Os10g33360*	expressed protein (drought-induced proteins, anther and pollen wall remodelling/metabolism proteins contribute to the tolerance of rice to salt stress)	Ke *et al.(2009)* Sarhadi *et al.*(2012)
K+ conc.	*qK1*	1	39922192-39923794	16	*LOC_Os01g68730*	RNA-binding protein FUS, putative, expressed (involved in cellular stress	Tischbein *et al.* (2019)

**Characters**	**QTL name**	**Chr.**	**Position (bp)**	**Number of locus**	**Candidate gene**	**Possible function**	**Reference**
						response, responses against pathogen infection).	Woloshen *et al.* (2011)
Na-K ratio	*qNaK12*	12	10048848-10053017	19	*LOC_Os12g17530*	expressed protein (drought-induced proteins, anther and pollen wall remodelling/metabolism proteins contribute to the tolerance of rice to salt stress)	Ke *et al.*(2009) Sarhadi *et al.*(2012)
