# Supplementary material for: Molecular Mapping to Discover Reliable Salinity-Resilient QTLs from the Novel Landrace Akundi in Two Bi-Parental Populations Using SNP-Based Genome-Wide Analysis in Rice
Source: Int J Mol Sci. 2023 Jul 6;24(13):11141. doi: 10.3390/ijms241311141 (PMC10342095; doi:10.3390/ijms241311141)
Supplement: Supplementary file 1 [file ijms-24-11141-s001.zip › ijms-2445956-supplementary/ijms-2445956-revised-supplementary/Supplementary Table S3.pdf]

Supplementary Table S3. Epistatic gene interaction for the traits related to seedling stage salinity tolerance in a $F_{2:3}$ population derived from the cross BR 28/ Akundi

Trait	Chr.1	Position1 (cM)	FM1	Chr. 2	Position2 (cM)	LOD	FM2	PVE (%)	Add1	Add2	Addby Add	TI
SES	3	96.9	M262-M265	4	70.7	10	M340-M341	1.251	0.112	-0.072	-0.092	Between complementary loci
	1	50.8	M34-M35	5	60.2	19	M419-M420	0.037	0.013	-0.013	-0.013	Between complementary loci
	3	61.9	M247-M248	6	26	23	M482-M483	0.659	-0.015	0.042	-0.521	Between complementary loci
	6	21	M478-M479	6	91	13	M511-M512	1.266	-0.101	-0.273	-0.803	Between complementary loci
	1	20.8	M17-M18	6	101	10	M514-M515	1.239	0.382	-0.485	0.601	Between complementary loci
	4	100.7	M357-M359	6	101	11	M514-M515	1.246	-0.372	-0.469	-0.616	Between complementary loci
	9	85.6	M732-M733	11	21	10	M793-M794	1.240	0.190	0.056	0.452	Between complementary loci
	6	26	M482-M483	11	31	9	M798-M799	1.260	0.980	-0.131	-0.132	Between complementary loci
Survival	5	50.2	M414-M415	5	110.2	8	M445-M446	0.778	0.529	0.194	-9.727	Between complementary loci
	5	50.2	M414-M415	9	10.6	6	M684-M685	0.737	-6.791	2.075	3.111	Between complementary loci
Shoot length	6	26	M482-M483	12	36.4	69	M892-M893	1.320	1.507	-1.552	-2.671	Between complementary loci
	3	31.9	M226-M227	12	76.4	51	M921-M922	1.527	4.915	1.564	1.599	Between complementary loci
	4	75.7	M343-M344	12	76.4	50	M921-M922	1.703	5.586	1.661	1.665	Between complementary loci
	10	40.6	M755-M756	12	76.4	51	M921-M922	1.544	-4.533	1.545	-1.590	Between complementary loci
	11	1	M787-M788	12	81.4	26	M924-M925	0.368	-0.645	0.171	-1.145	Between complementary loci

Trait	Chr.1	Position1 (cM)	FM1	Chr. 2	Position2 (cM)	LOD	FM2	PVE (%)	Add1	Add2	Addby Add	TI
Shoot dry weight	6	86	M510-M511	10	50.6	34	M764-M765	1.331	-0.001	0.002	0.002	Between QTLs and background
Root length	10	50.6	M764-M765	12	36.4	15	M892-M893	0.058	-0.298	-0.320	0.294	Between complementary loci
	1	125.8	M75-M76	2	90.4	15	M165-M166	0.806	0.194	0.080	0.097	Between complementary loci
	2	90.4	M165-M166	4	50.7	25	M329-M330	0.515	-0.023	-2.527	0.023	Between complementary loci
SPAD	2	90.4	M165-M166	6	121	32	M531-M532	0.924	-0.066	0.599	0.311	Between complementary loci
	2	90.4	M165-M166	9	30.6	22	M701-M702	0.248	2.018	-1.477	2.388	Between complementary loci
	2	90.4	M165-M166	10	60.6	15	M769-M770	0.623	-0.221	-2.972	0.197	Between complementary loci
Na^+ Conc.	2	110.4	M180-M181	7	97	11	M579-M580	1.012	0.004	0.017	-0.018	Between QTLs and background
K^+ Conc.	3	51.9	M240-M241	12	76.4	24	M921-M922	0.494	-0.073	-0.007	0.007	Between complementary loci
	4	10.7	M305-M306	4	110.7	11	M363-M364	0.848	-0.067	0.085	-0.015	Between complementary loci
	3	61.9	M247-M248	12	76.4	49	M921-M922	0.501	0.035	-0.029	0.029	Between complementary loci
NaK Ratio	4	75.7	M343-M344	12	76.4	39	M921-M922	0.984	-0.153	-0.035	-0.035	Between complementary loci
	9	35.6	M703-M704	12	76.4	42	M921-M922	1.114	0.154	-0.034	0.035	Between complementary loci

Chr. 1: Chromosome ID1; **FM1: Flanking markers (Position1); ***Chr. 2: Chromosome ID2;[1] FM2: Flanking markers (Position2);[2] LOD: LOD score due to epistatic effects;[3]PVE (%): PVE due to epistatic effects (%); Add1: Estimated additive effect of position1; Add2: Estimated additive effect of position 2; Add1 by Add2: Additive by additive epistatic at the two interacting positions; TI: Types of interaction
