# Supplementary material for: Molecular Mapping to Discover Reliable Salinity-Resilient QTLs from the Novel Landrace Akundi in Two Bi-Parental Populations Using SNP-Based Genome-Wide Analysis in Rice
Source: Int J Mol Sci. 2023 Jul 6;24(13):11141. doi: 10.3390/ijms241311141 (PMC10342095; doi:10.3390/ijms241311141)
Supplement: Supplementary file 1 [file ijms-24-11141-s001.zip › ijms-2445956-supplementary/ijms-2445956-revised-supplementary/Supplementary Table S4.pdf]

Supplementary Table S4 Comparisons of the QTL regions identified in this study for salinity tolerance at seedling stage, with previously mapped QTLs from different populations and for different growth stages

**Gene/QTL name**	**Chr.**	**Growth Stage**	**Mapping Population**	**Evaluation index**	**Interval/peak**	**Position (cM)**	**PVE (%)**	**References**	**Remarks**
QNa	1	Seedling	RIL	High Na uptake				Flowers et al. 2000 qNa2 (134.4 cM), qNa10 (68.8cM) – This study	Absent
Trait-based QTL	1	Seedling	RIL	Na^+ uptake	E12M55-3	74 cM	8.9	Koyama et al. 2001 *qNa2* (134.4 cM), *qNa10* (68.8cM) – This study	,,
Trait-based QTL	1	Seedling	RIL	K^+ concentration	E12M37-1	56 cM	10.6	Koyama et al. 2001 *qK1* (156.5 cM) – This study	Novel
Trait-based QTL	1	Seedling	RIL	Na: K ratio	E12M57-1	74 cM	9.1	Koyama et al. 2001 *qNaKR12* (39.4 cM) – This study	Absent
Saltol	1	Seedling	RIL	Na^+ uptake	RM140 - C1733S	between 51.6 and 65.9cM / 13.87 Mb	39.2	Bonilla et al. 2002 *qSUR11* (21 cM) – This study	Novel
Saltol	1	Seedling	RIL	K^+ uptake	RM140 - C1733S	between 51.6 and 65.9cM / 13.87 Mb	43.9	,, *qK1* (156.5 cM) – This study	,,
Saltol	1	Seedling	RIL	Na/K ratio	RM140 - C1733S	between 51.6 and 65.9cM / 13.87 Mb	43.2	,, *qNaKR12* (39.4 cM) – This study	,,
qSDW1	1	Seedling	BIL	Shoot dry weight	36.09-36.28	0.75 cM	28.6	Nakhla et al. 2021 *qSDW1* 123.4 cM), *qSDW10* (68.6) – This study	,,
qSDS-1	1	Seedling	$F_{2:3}$	Seedling survival	C813 - C86	-	18.0	Lin et al. 2004 *qSUR11* (21 cM) – This study	Novel
qSKC-1	1	Seedling	$F_{2:3}$	Shoot K^+ concentratio n	C1211 - S2139	-	40.1	,, *qK1* (156.5 cM) – This study	Absent

Gene/QTL name	Chr.	Growth Stage	Mapping Population	Evaluation index	Interval/peak	Position (cM)	PVE (%)	References	Remarks
qRNTQ-1	1	Seedling	$F_{2:3}$	Root Na^+ total quantity	C813 - C86	-	12.4	,,	,,
OsHKT1;5 (SKC1)	1	Seedling	Advanced backcross	Shoot K^+ concentration	K159 - K061	11.46 Mb	-	Ren et al. 2005 *qK1* (156.5 cM) – This study	Absent
qST1	1	Seedling	RIL		Est1-2 & RZ569A	40 cM	27.8	Lee et al. 2006	,,
qSES1	1	Seedling	F_2	SES	RM8094-RM582	-	19.6	Bimpong et al. 2014 *qSES1* (151.8 cM) – This study	Novel
qSur1.1	1	Seedling	F_2	Survival	RM472-RM14	162.9 cM	16.0	Rahman et al. 2019 *qSUR11* (21 cM) – This study	Novel
qSL1.1	1	Seedling	RIL	Shoot length	id1023892–id1024836	162.6 cM	20.6	Bizimana et al. 2017 *qSL1* (156 cM), *qSL8* (22 cM) – This study	Same
qSL1.2	1	Seedling	RIL	Shoot length	id1024972–id1025983	168.6 cM	11.8	,, *qSL1* (156 cM), *qSL8* (22 cM) – This study	Absent
qSIS1.39	1	Seedling	IL	Salt injury score (SIS)	RM3810	39.5 cM	6.4	De Leon et al. 2017	Novel
qSHL1.39	1	Seedling	IL	Shoot length	RM3810	39.5 cM	8.2	,, *qSL1* (156 cM), *qSL8* (22 cM) – This study	Same
qSHL1.41	1	Seedling	IL	Shoot length	RM5362	41.1 cM	6.8	,,	-
qK2	2	Seedling	BIL	K+ uptake	19.80-20.15	1.37 cM	26.1	Nakhla et al. 2021 *qK1* (156.5 cM) – This study	,,
qSHL2.3	2	Seedling	IL	Shoot length	RM211	3.0 cM	6.6	,,	Absent
qYLD2.1	2	Reproductive	F_2	Yield	Id2004774	40.1cM	14.6	Mondal et al. 2019	,,
qSES2.1	2	Seedling	RIL	SES	id2004774–id2007526	64.8 cM	11.1	Bizimana et al. 2017 *qSES1* (151.8 cM) – This study Mondal et al. 2019	,,
qNFS2.1	2	Reproducti	F_2	Filled		40.1 cM	15.3	,,	,,
qPFS2.1	2	ve	F_2	spikelet	id2004774	122.1 cM	18.4	Nakhla et al. 2021	,,
qNa2.1	2	Reproducti	BIL	number	id20133434	0.43 cM	5.3	,,	,,
		ve		Filled	4.15-4.26				
		Seedling		spikelet (%)				,, *qNa2* (134.4 cM), *qNa10*	
qNa2.2	2		BIL	Na^+ uptake		4.59 cM	17.8	(68.8cM) – This study	,,
					4.26-5.43				
		Seedling							
				Na^+ uptake					

Gene/QTL name	Chr.	Growth Stage	Mapping Population	Evaluation index	Interval/peak	Position (cM)	PVE (%)	References	Remarks
qSIS2.3	2	Seedling	IL	Salt injury score (SIS)	RM211	3.0 cM	6.5	,,	,,
qSHL2.3	2	Seedling	IL	Shoot length	RM211	3.0 cM	6.6	,, *qSL1* (156 cM), *qSL8* (22 cM) – This study	Absent
qST3	3	Seedling	RIL		RG179 - RZ596	138 cM	9.2	Lee et al. 2006	Novel
qSES3.1	3	Seedling	$F_{2:3}$	SES	RM5626-R3M53	111.0 cM	23.0	Rahman et al. 2019 *qSES1* (151.8 cM) – This study<br>,, *qSUR11* (21 cM) – This study	Novel
qSur3.2	3	Seedling	$F_{2:3}$	Survival	RM5626-R3M53	111.0	18.0		Novel
Q_{NaK}	4	Seedling	RIL	Na:K discrimination				Flowers et al. 2000 *qNaKR12* (39.4 cM) – This study	,,
Trait-based QTL	4	Seedling	RIL	K^+ uptake	E12M65-1	10 cM	6.8	Koyama et al. 2001<br>*qK1* (156.5 cM) – This study	Novel
Trait-based QTL	4	Seedling	RIL	K^+ concentration	E15M53-2	90 cM	8.8	,, *qK1* (156.5 cM) – This study	,,
	4	Seedling	RIL	Na^+ concentration	E12M73-1; E12M75-5; E15M50-5; E12M79-1	24 cM	6.7	,, *qNa2* (134.4 cM), *qNa10* (68.8cM) – This study	,,
Trait-based QTL	4	Seedling	RIL	Na:K ratio	E12M65-1	14 cM	9.6	,, *qNaKR12* (39.4 cM) – This study	,,
qRKC-4	4	Seedling	$F_{2:3}$	Root K^+ concentration	C891 - C513	-	21.6	Lin et al. 2004	,,
qSDM-5	5	Seedling	Double haploid lines	Seedling dry matter	RZ70-RZ225	-	17.9	Prasad et al. 2000 *qSDW1* 123.4 cM), *qSDW10* (68.6) – This study	,,
qSHL-5	5	Seedling	F2:4	Shoot length	RM13-RM164	106.8 cM	19.5	Ghomi et al. 2013 *qSL1* (156 cM), *qSL8* (22 cM) – This study	Absent
qSHL5.04	5	Seedling	IL	Shoot length	RM17749	0.4 cM	8.5	De Leon et al. 2017	,,
Q_{K1}	6	Seedling	NIL	K^+ uptake				Flowers et al. 2000	,,

Gene/QTL name	Chr.	Growth Stage	Mapping Population	Evaluation index	Interval/peak	Position (cM)	PVE (%)	References	Remarks
								qK1 (156.5 cM) – This study	
qSGEM-6	6	Germina tio n	Double haploid lines	Seed germination	RZ398-RG213	-	16.3	Prasad et al. 2000	,,
Trait-based QTL	6	Seedling	RIL	Dry mass	E12M55-2	34 cM	9.7	Koyama et al. 2001	Novel
Trait-based QTL	6	Seedling	RIL	K^+ uptake	OSR19; E12M80-2	30 cM	7.6	,,	Absent
Trait-based QTL	6	Seedling	RIL	Na^+ concentration	E12M35-2	106 cM	6.4	,, *qNa2* (134.4 cM), *qNa10* (68.8cM) – This study	,,
qSDS-6	6	Seedling	$F_{2:3}$	Seedling survival	C214 - R2549	-	17.0	Lin et al. 2004 *qSUR11* (21 cM) – This study	Novel
qSES6	6	Seedling	F2	SES	RM586-RM253	-	39.7	Bimpong et al. 2014 *qSES1* (151.8 cM) – This study	,,
qSHL-6	6	Seedling	F2:4	Shoot length	RM402-RM549	87.4 cM	14.6	Ghomi et al. 2013 *qSL1* (156 cM), *qSL8* (22 cM) – This study	,,
qSL6.1	6	Seedling	RIL	Shoot length	fd13–id6004343	18.5 cM	12.1	Bizimana et al. 2017 *qSL1* (156 cM), *qSL8* (22 cM) – This study	Novel
qSIS6.5	6	Seedling	IL	Salt injury score (SIS)	RM253	5.4 cM	7.0	De Leon et al. 2017	,,
qSHL6.5	6	Seedling	IL	Shoot length	RM253	5.4 cM	12.6	,,	Absent
qSDS-7	7	Seedling	$F_{2:3}$	Seedling survival	R2401 - L538T7	-	13.9	Lin et al. 2004 *qSUR11* (21 cM) – This study	,,
qSNC-7	7	Seedling	$F_{2:3}$	Shoot Na^+ concentration	C1057 - R2401	-	48.5	,,	Absent
qSNTQ-7	7	Seedling	$F_{2:3}$	Shoot Na^+ total quantity	C1057 - R2401	-	16.1	,,	,,
qRKC-7	7	Seedling	$F_{2:3}$	Root K^+ concentratio n	C1057 - R2401	-	17.8	,,	Absent
qRKTQ-7	7	Seedling	$F_{2:3}$	Root K^+ total quantity	C1057 - R2401	-	17.3	,,	,,
qSL7	7	Seedling	RIL	Higher shoot length	SNP7-191-SNP7-226	86.31 cM	9.9	Jahan et al. 2020	,,

Gene/QTL name	Chr.	Growth Stage	Mapping Population	Evaluation index	Interval/peak	Position (cM)	PVE (%)	References	Remarks
qSIS7.12	7	Seedling	IL	Salt injury score (SIS)	RM214	12.8 cM	5.9	De Leon et al. 2017	,,
qSIS7.17	7	Seedling	IL	Salt injury score (SIS)	RM5793	17.5 cM	8.1	,,	,,
qSHL7.12	7	Seedling	IL	Shoot length	RM214	12.8 cM	6.7	,, *qSL1* (156 cM), *qSL8* (22 cM) – This study	,,
Q_{K2}	9	Seedling	NIL	K^+ uptake				Flowers et al. 2000 *qK1* (156.5 cM) – This study	Absent
Trait-based QTL	9	Seedling	RIL	K^+ uptake	E12M55-4	96 cM	19.6	Koyama et al. 2001 *qK1* (156.5 cM) – This study	,,
qRNC-9	9	Seedling	$F_{2:3}$	Root Na^+ concentration	R1751 - R2638	-	16.7	Lin et al. 2004	,,
qSHL-9	9	Seedling	$F_{2:4}$	Shoot length	E37-M60-13-E36-M60-1	136.1 cM	9.9	Ghomi et al. 2013	,,
qSES10	10	Seedling	F_2	SES	RM228-RM333	-	30.7	Bimpong et al. 2014 *qSES1* (151.8 cM) – This study	Absent
qSHL-10	10	Seedling	$F_{2:4}$	Shoot length	RM2863-E36-M61-13	111.4 cM	4.1	Ghomi et al. 2013 *qSL1* (156 cM), *qSL8* (22 cM) – This study	,,
qSPAD10.1	10	Seedling	F_2	SPAD value	RM501C-RM5806	33.2 cM	15	Rahman et al. 2019 *qSPAD7* (91.4 cM) – This study	Novel
qSES11	11	Seedling	F_2	SES	RM536-RM287	-	37.2	Bimpong et al. 2014 *qSES1* (151.8 cM) – This study Mondal et al.2019	,,
qTS11.1	11	Reproductive	F_2	Total spikelet number	Id11000858	14.0 cM	15.8	Nakhla et al. 2021 *qK1* (156.5 cM) – This study	,,
q K11	11	Seedling	BIL	K^+ uptake	25.75-2.59	.43 cM	9.6		,,
qSL12.1	12	Seedling	RIL	Shoot length	id12000252–id12001321	6.9 cM	9.8	Bizimana et al. 2017 *qSL1* (156 cM), *qSL8* (22 cM) – This study	Novel
qSES12.1	12	Seedling	RIL	SES	id12000252-id12001321	6.9 cM	10.6	O'Connor et al. 2020 *qSES1* (151.8 cM) – This study	,,
qDEG-S-2-2		Reproductive	Backcross	Spikelet degeneration		6.8 cM	34.44	,,	,,
qDEG-S-4-3			Backcross			4.19 cM	17.43	Rahman et al. 2019 *qSUR11* (21	,,
qSur12.3	12		$F_{2:3}$			67.1 cM	16.0	cM) – This study	Novel

Gene/QTL name	Chr.	Growth Stage	Mapping Population	Evaluation index	Interval/peak	Position (cM)	PVE (%)	References	Remarks
		Reproductive		Spikelet degeneration	S12055-RM28466				
		Seedling		Survival					

Chr: Chromosome number; IL: Introgression line; PVE: Phenotypic variation explained; RIL: Recombinant inbred line; BIL: Backcross Introgression line; SES: Overall phenotypic performance; SL: Shoot length; Na: Na^{+}: concentration; K: K^{+}: concentration; NaK-R: Na-K ratio, Sur: Survival; indicates embedded QTLs identified in this study
